# Occupational safety and health aspects of corporate social responsibility reporting in Japan from 2004 to 2012

**DOI:** 10.1186/s12889-017-4356-y

**Published:** 2017-05-02

**Authors:** Tomohisa Nagata, Akinori Nakata, Koji Mori, Takashi Maruyama, Futoshi Kawashita, Masako Nagata

**Affiliations:** 1 Department of Occupational Health Practice and Management, Institute of Industrial Ecological Sciences, University of Occupational and Environmental Health, 1-1 Iseigaoka, Yahatanishi-ku, Kitakyushu city, Kitakyushu, 807-8555 Japan; 2 Department of Occupational and Community Health Nursing, School of Health Sciences, University of Occupational and Environmental Health, Kitakyushu-city, Fukuoka Japan; 3 Occupational Health Training Center, University of Occupational and Environmental Health, Kitakyushu-city, Fukuoka Japan; 4 Department of Physiology, School of Medicine, University of Occupational and Environmental Health, Kitakyushu-city, Fukuoka Japan

**Keywords:** Reporting, Corporate social responsibility, Occupational safety and health, Yearly trend, Category of industry, Size of company

## Abstract

**Background:**

A number of companies publish corporate social responsibility (CSR) reporting in booklets and other publicly available formats. The purpose of this paper is to clarify the nine-year (2004–2012) trend of occupational safety and health (OSH) activities as described in CSR reporting (by industry sector and company size).

**Methods:**

We investigated CSR reporting on the website in all Japanese companies listed on the first section of the Tokyo Stock Exchange. The data were extracted from CSR reporting of each company every year from 2004 to 2012. We counted the pages dedicated to information on OSH activities by industry sector and company size and calculated the rate of OSH divided by total CSR-related activities.

**Results:**

The number of companies publishing CSR reports increased in all industry sectors, although the rate of inclusion of OSH activity within CSR reports increased only among sectors such as construction, manufacturing, transportation, and commerce. Among all company size, CSR reporting increased constantly throughout all observed years. The proportion of companies that had described OSH in CSR reporting increased from 2004 to 2012, and 76.5% companies had described OSH activities in 2012. The average number of pages of CSR-related report was 34.2 in 2004, increasing to 43.1 in 2012. The proportion of described pages of OSH activities in total CSR reporting increased gradually, and 2.7% in 2012. The focus of CSR reporting gradually shifted from ‘environment’ to ‘social activity including OSH’.

**Conclusions:**

Majority of companies are putting more emphasis on OSH in CSR reporting in Japan.

## Background

Corporate social responsibility (CSR) is defined by the European Commission as a concept whereby companies integrate social and environmental concerns into their business operations and interaction with their stakeholders on a voluntary basis [1]. In developed countries, the concept of CSR has developed tremendously during the past decade, leading the International Standardized Organization (ISO) to publish ISO 26000 in 2010 [2]. ISO 26000:2010 has seven core subjects, one being labor practices. Labor practices include health and safety at work, which is related to the promotion and maintenance of the highest degree of physical, mental and social wellbeing of workers and the prevention of harm to health caused by working conditions. CSR is connected to occupational safety and health (OSH) in research practices [3, 4]. OSH is an important aspect of CSR, as OSH is one of the indicators used to measure companies’ overall progress in CSR [5].

A number of companies publish CSR-related reports in booklets and other publicly available formats such as portable document format (PDF) files, which can be obtained from company website. This accountability mechanism requires the ongoing implementation of CSR activities [6]. Various guidelines for reporting CSR activities have been published, including the influential guidelines of the Global Reporting Initiative (GRI), first launched in 2000 [7]. GRI version 4 (G4) is the latest version of the GRI’s Sustainability Reporting Guidelines [8]. Many companies use this document as a reference for developing CSR-related reports. GRI Guidelines recommend to describe OSH activities such as G4-LA5: Percentage of total workforce represented in formal joint management–worker health and safety committees that help monitor and advise on occupational health and safety programs, G4-LA6: Type of injury and rates of injury, occupational diseases, lost days, and absenteeism, and total number of work-related fatalities, by region and by gender, G4-LA7: Workers with high incidence or high risk of diseases related to their occupation, G4-LA8: Health and safety topics covered in formal agreements with trade unions, and G2-LA14: Evidence of substantial compliance with the ILO Guidelines for Occupational Health Management Systems.

According to the most recent review including 53 studies about CSR reporting [9], almost all studies analyze only tens of corporate reports. From this small number, it may be difficult to generalize and grasp entire trends and transitions in CSR reporting. This limited analysis likewise applies to CSR reporting focusing on OSH [4]. Few studies analyze yearly trends, which limits the ability to track industry dynamism.

The purpose of this study is to investigate and analyze CSR-related reports according to category of industry and size of company (number of employees) from 2004 to 2012 in all Japanese companies listed in the first section of the Tokyo Stock Exchange (TSE First Section) to understand the trends and development of OSH activities in CSR-related reports.

## Methods

### Data sources

The subjects of this study were the companies listed on the TSE First Section on October 15th each year from 2004 to 2012. The company list was obtained from the autumn catalogue of the Japanese company quarterly journal “Kaisha Shikiho” [10]. We listed the company names, categories of industry, and sizes of companies from the Kaisha Shikiho each year of the study period, gathering company data from each company’s formal announcement.

### Investigation

Investigations were carried out every year from 2004 to 2012 by several of our researchers during February and March of the following year. First, we investigated whether or not each company had published a CSR-related report using a PDF file and brochure over the internet. We considered CSR-related publications to be reports if they were eight or more pages long, including front and back covers. Second, each report was classified by its title; titles included environmental report, environmental and social report, CSR report, sustainability report, and others (including responsible care report). Third, we classified all pages of each report as referring to environmental activity, social activity, or other classifications. We found OSH most often in sections on social activity. We investigated OSH items like policy on OSH, health and safety committees, type and rates of injury, formal agreements on OSH with trade unions, occupational safety and health management system, countermeasure for mental health, and so on.

### Quality control of the investigation

We conducted the investigation year by year and developed evaluation standards, producing a manual of protocols for investigation of CSR-related reports. Before starting the analysis, we investigated the same reports independently as a trial and shared the results of our analyses. If the results were not perfectly matched, we discussed the discrepancy, identified the cause and made modifications. This process was conducted each year of our investigation.

### Statistical analysis

We calculated two proportions for companies each year from 2004 to 2012 by category of industry and by size of company (number of employees). One is the proportion of companies of the TSE First Section that published CSR-related reports (the published rate), and the other is the proportion of companies publishing CSR-related reports that described OSH activities (the described rate). Yearly changes from 2004 to 2012 were analyzed using the Cochran-Armitage test for trends in proportions. Calculation of the proportions was performed using IBM SPSS Statistics, Version 22.0 (IBM Corp., Chicago, IL, USA) and statistical analysis was performed using STATA/SE 13.1 software for windows (STATA Corp., College Station, Texas, USA). *P*-values of less than 0.05 were considered statistically significant.

### Ethics

We used only publicly available information in the study and did not use personally identifiable information.

## Results

Descriptions of the Japanese companies listed in the TSE First Section by category of industry and size of company (number of employees) are summarized in Table 1. The number of companies in the TSE First Section in Japan increased from 1583 in 2004 to 1736 in 2008 and decreased to 1717 in 2012. The number of companies, led by ‘mining’, ‘transportation, information and communication’, ‘commerce’, ‘real estate’ and ‘services’, increased by more than 10% from 2004 to 2012.

**Table 1:** Description of all Japanese companies listed on the first section of the Tokyo Stock Exchange by category of industry and size of company in 2004–2012

	2004		2005		2006		2007		2008		2009		2010		2011		2012		Increase rate during nine years
	N	%	N	%	N	%	N	%	N	%	N	%	N	%	N	%	N	%	
Total	1583		1661		1705		1723		1736		1734		1707		1702		1717		
Category of industry																			
Fisheries, agriculture and forestry	6	0.4	6	0.4	6	0.4	6	0.3	5	0.3	5	0.3	5	0.3	5	0.3	5	0.3	83.3%
Mining	6	0.4	7	0.4	6	0.4	6	0.3	6	0.3	6	0.3	7	0.4	7	0.4	7	0.4	116.7%*
Construction	104	6.6	105	6.3	105	6.2	104	6.0	105	6.0	103	5.9	100	5.9	98	5.8	97	5.6	93.3%
Manufacturing	821	51.9	842	50.7	853	50.0	860	49.9	856	49.3	861	49.7	845	49.5	840	49.4	844	49.2	102.8%
Electricity and gas	16	1.0	17	1.0	17	1.0	17	1.0	17	1.0	17	1.0	17	1.0	17	1.0	17	1.0	106.3%
Transportation, information and communication	138	8.7	145	8.7	155	9.1	158	9.2	166	9.6	169	9.7	165	9.7	166	9.8	169	9.8	122.5%*
Commerce	248	15.7	272	16.4	282	16.5	285	16.5	279	16.1	285	16.4	291	17.0	298	17.5	300	17.5	121.0%*
Finance and insurance	140	8.8	145	8.7	151	8.9	146	8.5	152	8.8	142	8.2	135	7.9	128	7.5	129	7.5	92.1%
Real estate	37	2.3	45	2.7	49	2.9	52	3.0	54	3.1	49	2.8	46	2.7	47	2.8	48	2.8	129.7%*
Services	67	4.2	77	4.6	81	4.8	89	5.2	96	5.5	97	5.6	96	5.6	96	5.6	101	5.9	150.7%*
Size of company																			
-49	41	2.6	51	3.1	48	2.8	52	3.0	51	2.9	59	3.4	66	3.9	72	4.2	70	4.1	170.7%*
50–299	190	12.0	219	13.2	240	14.1	243	14.1	257	14.8	266	15.3	263	15.4	265	15.6	274	16.0	144.2%*
300–999	562	35.5	602	36.2	624	36.6	631	36.6	606	34.9	599	34.5	570	33.4	575	33.8	594	34.6	105.7%
1000–2999	518	32.7	522	31.4	528	31.0	526	30.5	524	30.2	523	30.2	523	30.6	520	30.6	501	29.2	96.7%
3000–4999	124	7.8	123	7.4	112	6.6	122	7.1	129	7.4	135	7.8	136	8.0	131	7.7	133	7.7	107.3%
5000–9999	92	5.8	86	5.2	91	5.3	89	5.2	84	4.8	84	4.8	77	4.5	71	4.2	69	4.0	75.0%
10,000-	52	3.3	54	3.3	56	3.3	55	3.2	60	3.5	61	3.5	64	3.7	56	3.3	59	3.4	113.5%*
uncertain	4	0.3	4	0.2	6	0.4	5	0.3	25	1.4	7	0.4	8	0.5	12	0.7	17	1.0	

*N* Number of the companies by category of industry and by size of company by number of employees

^*^Those which increased more than 10% from 2004 to 2012

### Analysis by category of industry

The number and proportion of the companies publishing CSR-related reports and the companies that described OSH activities in CSR-related reports by category of industry from 2004 to 2012 are shown in Table 2. The number of companies that published CSR-related reports in 2004 was 413 (26.1%); this figure increased to 663 (38.6%) in 2012. The number of companies in all categories of industry except ‘electricity and gas’ increased from 2004 to 2012. The number of companies that described OSH activities was 211 (51.1%) in 2004, increasing to 507 (76.5%) in 2012. The companies classified as ‘construction’, ‘manufacturing’, ‘transportation, information and communication’, and ‘commerce’ showed an upward trend from 2004 to 2012, but the others did not show such a tendency.

**Table 2:** The companies which published corporate social responsibility (CSR) related reports divided by total number of companies listed on the first section of the Tokyo Stock Exchange by category of industry in 2004–2012

	2004			2005			2006			2007			2008			2009			2010			2011			2012			
	n(CSR)	n(CSR)/N	(%)	n	n(CSR)/N	(%)	n	n(CSR)/N	(%)	n	n(CSR)/N	(%)	n	n(CSR)/N	(%)	n	n(CSR)/N	(%)	n	n(CSR)/N	(%)	n	n(CSR)/N	(%)	n	n(CSR)/N	(%)	*P* for trend
Total	413	26.1		494	29.7		569	33.4		555	32.2		572	32.9		609	35.1		625	36.6		619	36.4		663	38.6		*p* < .001
Category of industry																												
Fisheries, agriculture and forestry	0	0.0		2	33.3		1	16.7		2	33.3		3	60.0		3	60.0		3	60.0		3	60.0		3	60.0		*p* < .001
Mining	0	0.0		1	14.3		1	16.7		2	33.3		3	50.0		3	50.0		3	42.9		3	42.9		3	42.9		*p* < .05
Construction	24	23.1		36	34.3		42	40.0		39	37.5		46	43.8		54	52.4		56	56.0		53	54.1		55	56.7		*p* < .001
Manufacturing	303	36.9		356	42.3		403	47.2		386	44.9		388	45.3		412	47.9		414	49.0		397	47.3		427	50.6		*p* < .001
Electricity and gas	15	93.8		16	94.1		15	88.2		16	94.1		16	94.1		17	100.0		15	88.2		14	82.4		14	82.4		n.s.
Transportation, information and communication	19	13.8		23	15.9		31	20.0		29	18.4		36	21.7		40	23.7		34	20.6		37	22.3		43	25.4		*p* < .01
Commerce	38	15.3		37	13.6		46	16.3		41	14.4		46	16.5		42	14.7		52	17.9		59	19.8		66	22.0		*p* < .01
Finance and insurance	11	7.9		15	10.3		22	14.6		30	20.5		24	15.8		25	17.6		30	22.2		33	25.8		29	22.5		*p* < .001
Real estate	2	5.4		5	11.1		4	8.2		6	11.5		4	7.4		6	12.2		8	17.4		9	19.1		9	18.8		*p* < .05
Services	1	1.5		3	3.9		4	4.9		4	4.5		6	6.3		7	7.2		10	10.4		11	11.5		14	13.9		*p* < .001
	n(OSH)	n(OSH)/n(CSR)	(%)	n(OSH)	n(OSH)/n(CSR)	(%)	n(OSH)	n(OSH)/n(CSR)	(%)	n(OSH)	n(OSH)/n(CSR)	(%)	n(OSH)	n(OSH)/n(CSR)	(%)	n(OSH)	n(OSH)/n(CSR)	(%)	n(OSH)	n(OSH)/n(CSR)	(%)	n(OSH)	n(OSH)/n(CSR)	(%)	n(OSH)	n(OSH)/n(CSR)	(%)	*P* for trend
Total	211	51.1		286	57.9		395	69.4		379	68.3		423	74.0		448	73.6		482	77.1		458	74.0		507	76.5		*p* < .001
Category of industry																												
Fisheries, agriculture and forestry				1	50.0		0	0.0		1	50.0		2	66.7		2	66.7		2	66.7		1	33.3		2	66.7		n.s.
Mining				1	100.0		1	100.0		2	100.0		2	66.7		3	100.0		2	66.7		3	100.0		3	100.0		n.s.
Construction	8	33.3		13	36.1		23	54.8		22	56.4		28	60.9		37	68.5		44	78.6		42	79.2		47	85.5		*p* < .001
Manufacturing	176	58.1		215	60.4		296	73.4		297	76.9		304	78.4		313	76.0		322	77.8		306	77.1		337	78.9		*p* < .001
Electricity and gas	8	53.3		11	68.8		12	80.0		10	62.5		9	56.3		13	76.5		12	80.0		10	71.4		12	85.7		n.s.
Transportation, information and communication	7	36.8		13	56.5		19	61.3		15	51.7		30	83.3		36	90.0		31	91.2		32	86.5		37	86.0		*p* < .001
Commerce	7	18.4		19	51.4		28	60.9		24	58.5		25	54.3		23	54.8		38	73.1		39	66.1		42	63.6		*p* < .001
Finance and insurance	4	36.4		9	60.0		11	50.0		4	13.3		17	70.8		15	60.0		22	73.3		15	45.5		14	48.3		n.s.
Real estate	1	50.0		2	40.0		3	75.0		2	33.3		3	75.0		3	50.0		4	50.0		6	66.7		7	77.8		n.s.
Services	0	0.0		2	66.7		2	50.0		2	50.0		3	50.0		3	42.9		5	50.0		4	36.4		6	42.9		n.s.

*n.s.* Not significant

*nCSR* number of companies which published CSR-related reports

*N* total number of companies listed in TSE1

*n(CSR)* number of the companies which published CSR-related reports

*n(OSH)* number of the companies that described occupational safety and health activities in CSR-related reports

### Analysis by company size

The number and proportion of the companies publishing CSR-related reports and the companies describing OSH activities in CSR-related reports by company size from 2004 to 2012 are shown in Table 3. CSR reporting increased among companies with between 50 and 9999 employees throughout the observation period. This upward trend was especially strong in the companies with between 300 and 9999 employees. The number of companies that described OSH activities increased among companies of all sizes throughout the observation period. This upward trend was especially strong among companies with between 50 and 2999 employees.

**Table 3:** The companies which published corporate social responsibility (CSR) related reports divided by total number of companies listed on the first section of the Tokyo Stock Exchange by size of company in 2004–2012

	2004			2005			2006			2007			2008			2009			2010			2011			2012			
	n(CSR)	n(CSR)/N	(%)	n	n(CSR)/N	(%)	n	n(CSR)/N	(%)	n	n(CSR)/N	(%)	n	n(CSR)/N	(%)	n	n(CSR)/N	(%)	n	n(CSR)/N	(%)	n	n(CSR)/N	(%)	n	n(CSR)/N	(%)	*P* for trend
Size of company by number of employees																												
-49	3	7.3		9	17.6		8	16.7		4	7.7		3	5.9		6	10.2		8	12.1		12	16.7		15	21.4		0.134
50–299	20	10.5		37	16.9		25	10.4		31	12.8		36	14.0		32	12.0		37	14.1		40	15.1		51	18.6		*p* < .05
300–999	75	13.3		148	24.6		135	21.6		131	20.8		134	22.1		145	24.2		142	24.9		133	23.1		152	25.6		*p* < .001
1000–2999	145	28.0		164	31.4		213	40.3		199	37.8		197	37.6		214	40.9		228	43.6		229	44.0		241	48.1		*p* < .001
3000–4999	67	54.0		59	48.0		71	63.4		78	63.9		84	65.1		89	65.9		90	66.2		90	68.7		91	68.4		*p* < .001
5000–9999	59	64.1		37	43.0		66	72.5		64	71.9		61	72.6		67	79.8		58	75.3		57	80.3		50	72.5		*p* < .001
10,000-	43	82.7		38	70.4		50	89.3		46	83.6		52	86.7		55	90.2		58	90.6		47	83.9		52	88.1		0.051
uncertain	1	25.0		2	50.0		1	16.7		2	40.0		5	20.0		1	14.3		4	50.0		11	91.7		11	64.7		
	n(OSH)	n(OSH)/n(CSR)	(%)	n(OSH)	n(OSH)/n(CSR)	(%)	n(OSH)	n(OSH)/n(CSR)	(%)	n(OSH)	n(OSH)/n(CSR)	(%)	n(OSH)	n(OSH)/n(CSR)	(%)	n(OSH)	n(OSH)/n(CSR)	(%)	n(OSH)	n(OSH)/n(CSR)	(%)	n(OSH)	n(OSH)/n(CSR)	(%)	n(OSH)	n(OSH)/n(CSR)	(%)	*P* for trend
Size of company by number of employees																												
-49	2	66.7		2	22.2		4	50.0		4	100.0		2	66.7		5	83.3		5	62.5		8	66.7		12	80.0		*p* < .05
50–299	7	35.0		22	59.5		13	52.0		20	64.5		25	69.4		24	75.0		29	78.4		29	72.5		40	78.4		*p* < .001
300–999	26	34.7		76	51.4		78	57.8		77	58.8		86	64.2		97	66.9		101	71.1		89	66.9		101	66.4		*p* < .001
1000–2999	69	47.6		92	56.1		145	68.1		129	64.8		147	74.6		157	73.4		172	75.4		169	73.8		182	75.5		*p* < .001
3000–4999	37	55.2		39	66.1		55	77.5		60	76.9		64	76.2		66	74.2		70	77.8		64	71.1		74	81.3		*p* < .01
5000–9999	37	62.7		27	73.0		51	77.3		49	76.6		53	86.9		54	80.6		51	87.9		46	80.7		42	84.0		*p* < .01
10,000-	33	76.7		26	68.4		48	96.0		39	84.8		41	78.8		45	81.8		51	87.9		43	91.5		47	90.4		*p* < .05
uncertain	0	0.0		2	100.0		1	100.0		1	50.0		5	100.0		0	0.0		3	75.0		10	90.9		9	81.8		-

*N* total number of companies listed in TSE1

*n(CSR)* number of the companies which published CSR-related reports

*n(OSH)* number of the companies that described occupational safety and health activities in CSR-related reports

### The titles and lengths of CSR-related reports

**Fig. 1:**
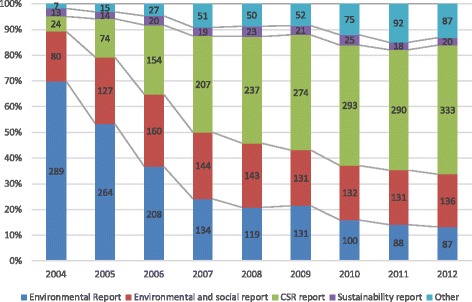
Detail on the titles of CSR-related reports from 2004 to 2012

The titles of CSR-related reports (environmental report, environmental and social report, CSR report, sustainability report and others) are shown in Fig. 1. Overall, 289 companies (70.0%) in 2004 used ‘environmental report’ as their report title, whereas 87 companies (13.1%) in 2012 used the same wording. In contrast, 24 companies (5.8%) in 2004 used ‘CSR report’ as the report title, whereas 333 companies (50.2%) in 2012 used the same wording.

The number of pages by content type is shown in Table 4. The average number of pages was 34.2 in 2004, increasing to 43.1 in 2012. The total number of pages was divided into three parts: environment activity, social activity (including OSH), and others (not environment activity, not social activity). The proportion of pages dedicated to environment activity decreased, while those that contained social activity (including OSH) and others increased.

**Table 4:** Mean page count of corporate social responsibility (CSR) related reports and the percentage of pages of each report content (environment, social activity, and other) in all companies listed on the first section of the Tokyo Stock Exchange in 2004–2012

Year	2004	2005	2006	2007	2008	2009	2010	2011	2012	*P* for trend
Total pages (mean)	34.19	34.08	37.48	39.27	39.72	38.65	40.09	40.66	43.05	
Proportion (%) of described pages in total										
Environment activity	66.8	59.1	53.4	40.2	35.9	35.7	34.9	31.7	30.5	*p* < .001
Social activity	14.0	14.0	20.2	16.7	20.2	16.9	19.6	16.8	23.5	*p* < .001
Occupational safety and health^a^	1.6	2.1	3.2	2.9	3.1	3.4	3.9	3.3	2.7	*p* < .001
Other (not environment, not social activity)	19.2	26.9	26.4	43.1	43.9	47.5	45.6	51.5	46.0	*p* < .001

^a^Occupational safety and health is a part of social activity

## Discussion

The purpose of this study was to investigate and analyze CSR-related reports by category of industry and size of company (number of employees) from 2004 to 2012 in all Japanese companies listed on the TSE First Section, to understand the trends and developments of CSR-related reports. We focused on OSH activities because they have to date received scant attention, although OSH is an important aspect of CSR. Key findings of this study were that the focus of CSR reporting in Japan gradually shifted from environment to social activity including OSH, and that the number of companies describing OSH in CSR reporting increased from 2004 to 2012.

The publication proportion of CSR-related reports increased in all categories except in ‘electricity and gas’, suggesting a growing awareness of CSR across all sectors. Of the ‘electricity and gas’ companies, 93.8% already published reports in 2004, and this trend was maintained through 2012 because of the sector’s importance in civilian life. In tertiary industries such as services, the awareness of CSR was not as high as that in primary and secondary industries. In terms of the company size by number of employees, the larger the size, the higher the publication proportion of CSR-related reports was every year from 2004 to 2012, showing that larger companies exhibited a higher interest in CSR. Independent of company size, the publication proportion of CSR-related reports increased from 2004 to 2012.

Changes in the social environment from 2004 to 2012 led to this upward trend. One factor was economic circumstances, but the publication rate of CSR-related reports may have little influence on a company’s economic circumstances. Economic fluctuations, as represented by Lehman’s collapse in September 2008, had significant impact on business and financial conditions across the majority of industrial sectors. After Lehman’s fall, there was no decrease in the number of companies publishing reports. This tendency may be attributed to two reasons. First, the companies that had already published CSR-related reports did not withdraw from publishing even if business conditions had deteriorated. Second, business and financial conditions may not have a direct impact on CSR reporting. Such speculation needs to be confirmed by further research.

The majority of report titles have recently changed from ‘Environmental report’ to ‘CSR report’ (Fig. 1), as observed in a past study [11]. The proportion of ‘Environmental reports’ among all CSR-related reports fell from 70.0% in 2004 to 13.1% in 2012, whereas ‘CSR reports’ increased from 5.8% in 2004 to 50.2% in 2012. In Japan, Environmental Reporting Guidelines were published in 1997 [12]. The change of the report title indicates that companies may have come to give higher value to social aspects of CSR.

OSH activities are labor practices and are described as social activity in CSR-related reports. The proportion of companies describing OSH activities among those publishing CSR-related reports (the described rate) increased yearly from 2004 to 2012, and the rate was 76.5% in 2012. By industry category, the described rate was more than 80% in 2012 in ‘mining’, ‘construction’, ‘electricity and gas’, and ‘transportation, information and communication’. The change of the described rate from 2004 to 2012 was enormous, especially in ‘construction’ (33.3% in 2004 to 85.5% in 2012) and in ‘transportation, information and communication’ (36.8% in 2004 to 86.0% in 2012). The accident severity rate in ‘construction’ and the rate of work time lost to injuries in ‘transportation, information and communication’ were higher in 2012 than in 2004 [13]. However, the described rate was less than 50% in 2012 in ‘finance and insurance’, and ‘services’. This suggests that OSH activities were not considered part of CSR in these categories of industry. The 12th Occupational Safety & Health Program of Ministry of Health, Labour and Welfare in Japan observed that occupational accidents increased by 16.7% in tertiary industries from 2002 to 2011; there were 43,053 casualties (worker’s death, illness or injuries due to a job-related reason with absence of 4 days or more) reported in 2002 and 50,243 in 2011 [14]. These data suggest that OSH activities need special attention in such sectors like ‘finance and insurance’ and ‘services’.

Considering the size of company by number of employees, the described rate of OSH in CSR-related reports increased from 2004 to 2012, to more than 65% in 2012 among companies of all sizes. The frequency and severity of work time lost to injuries and accidents was higher in smaller-sized companies [13], whereas the number of accidents and injuries was higher in larger companies.

In regard to OSH process, the Global Reporting Initiative (GRI) guidelines recommend to describe Occupational Safety and Health Management Systems (OSHMS) (G2-LA14) [15]. The Ministry of Health, Labour and Welfare in Japan also promotes OSH activities based on OSHMS. 47 companies (22.3%) described OSHMS in CSR reporting in 2004, and 157 companies (31.0%) did in 2012.

Internationally, the GRI developed reporting guidelines in 2000 [7], and updated the indicators as G2 in 2002 [15], G3 in 2006 [16], and G4 in 2013 [8]; all GRI guidelines recommend including OSH activities. In Japan, however, no guidelines mention describing OSH sections in CSR reporting. Overall, 76.5% of companies in Japan in 2012 described OSH activities in their CSR reporting, and 75.3% of the OSH descriptions included mental health activities for employees. Although the burden of mental disorders increased between 1990 and 2010 and mental health is one of the major issues around the world [17], the most recent guidelines do not clearly describe mental health activities.

### Strengths and limitations

This study has several strengths and limitations. This is one of the first studies to clarify the trend of OSH activities in CSR reporting from 2004 to 2012. To our knowledge, there are no studies that analyze the OSH aspect of CSR-related reports to reveal a 9-year trend. Second, our study checked all CSR-related reports and PDF files downloaded from the websites of Japanese companies listed on the TSE First Section from 2004 to 2012. Some companies disclosed CSR information only on their website and not through CSR-related reports or PDF files. In 2012, 633 companies had published reports of at least six pages, and 859 companies had published CSR information in paper format, as PDF files or as text on their website. Internet-based reporting became more popular because it is a cost effective means of disseminating corporate information [9].

There are at least three limitations to our study. First, although the concept of CSR includes accountability of CSR activities for different stakeholders, the reports do not describe all CSR activities. Second, we analyzed reporting only by companies listed on the TSE First Section, the leading companies in Japan. To be able to generalize our results, further analysis including small- and medium-sized companies would need to be undertaken. Third, the companies in this study include a few that were not listed continuously on the First Section of the TSE every year from 2004 to 2012 (5.4% of the companies in the First Section of the TSE from 2004 to 2012).

## Conclusions

This study discloses that about 40% of the companies listed on the TSE First Section have published CSR-related reports and that the percentage of companies publishing CSR reports increased from 2004 to 2012 regardless of industry sector. CSR reporting increased yearly among the companies with between 50 and 9999 employees.

This study demonstrates that about 75% of the companies publishing CSR-related reports describe OSH activities, and the described rate increased yearly from 2004 to 2012. Employees are important stakeholders for companies and are fundamental human capital in resource management. For other stakeholders like stockholders, the information about OSH activities for employees is useful in judging the company’s situation. While our results highlight OSH in CSR reporting, the next step is to analyze the content of reports in more detail.

## Abbreviations

CSR: Corporate social responsibility
GRI: Global reporting initiative
OSH: Occupational safety and health
TSE First Section: the First section of the Tokyo Stock Exchange
